# The Clinical Application of Pulsed Radiofrequency Induces Inflammatory Pain via MAPKs Activation: A Novel Hint for Pulsed Radiofrequency Treatment

**DOI:** 10.3390/ijms222111865

**Published:** 2021-11-01

**Authors:** Feng-Yen Lin, Kuo-Feng Huang, Jui-Chieh Chen, Meng-Fu Lai, Kuo-Hsing Ma, Chun-Chang Yeh

**Affiliations:** 1 Department of Internal Medicine and Taipei Heart Institute, Taipei Medical University, Taipei 110, Taiwan; g870905@tmu.edu.tw (F.-Y.L.); kfhuang@mail.chimei.org.tw (K.-F.H.); 2 Division of Cardiology, Taipei Medical University Hospital, Taipei 110, Taiwan; 3 Department of Obstetrics and Gynecology, Chi Mei Medical Center, Tainan 710, Taiwan; 4 Department of Biochemical Science and Technology, National Chiayi University, Chiayi 600, Taiwan; jcc@mail.ncyu.edu.tw; 5 Department of Anesthesiology, Tri-Service General Hospital, National Defense Medical Center, Taipei 114, Taiwan; mengfulai@gmail.com; 6 Department of Biology and Anatomy, National Defense Medical Center, Taipei 114, Taiwan; kuohsing91@yahoo.com.tw

**Keywords:** pulsed radiofrequency, pain, mitogen-activated protein kinases

## Abstract

Pulsed radiofrequency (PRF) works by delivering short bursts of radiofrequency to a target nerve, thereby affecting nerve signal transduction to reduce pain. Although preliminary clinical investigations have shown that PRF treatment can be used safely as an alternative interventional treatment in patients with refractory pain conditions, unexpected damage to a normal nerve/ganglion is still one of the possible complications of using the PRF strategy. Noxious pain may also be triggered if PRF treatment accidentally damages an intact nerve. However, few studies in the literature have described the intracellular modifications that occur in neuronal cells after PRF stimulation. Therefore, in this study, we evaluated the effects of PRF on unimpaired nerve function and investigated the potential mechanisms of PRF-induced pain. Wistar rats were stimulated with 30–60 V of PRF for 6 min, and mechanical allodynia, cold hypersensitivity, cytokine and matrix metalloproteinase (MMP) production, and mitogen-activated protein kinase activity (p38 MAPK, ERK1/2, JNK/SAPK) were analyzed. The results indicated that PRF stimulation induced a significant algesic effect and nociceptive response. In addition, the protein array and Western blotting analyses showed that the clinical application of 60 V of PRF can induce the activation of MAPKs and the production of inflammatory cytokines and MMPs in the lumbar dorsal horn, which is necessary for nerve inflammation, and it can be suppressed by MAPK antagonist treatment. These results indicate that PRF stimulation may induce inflammation of the intact nerve, which in turn causes inflammatory pain. This conclusion can also serve as a reminder for PRF treatment of refractory pain.

## 1. Introduction

In the past, clinicians often performed surgeries to remove the anatomical factors that cause pain or manage pain with pain-controlling drugs. However, many factors cause neuropathic pain, and there are complex relationships among these factors. Therefore, many intractable pains are very difficult to control, and refractory pain can harm the patients’ quality of life. Common clinical pains such as post-amputation stump pain, cervicogenic headache, cervical and lumbar radicular pain, knee and shoulder arthropathic pain, and neuropathic pain are intractable and difficult to control; intervention by one strategy alone often fails to achieve the expected effect, and even causes other systemic damages. In the last 20 years, interventional pain management specialists have begun to use pulsed radiofrequency (PRF) to manage refractory pain. PRF treatment mainly involves introducing heat generated by electromagnetic waves with a frequency of 500 kHz (temperature below 42 °C) and pulse width of 20 ms, 0.5 cm proximal to the division of the peripheral nerve or dorsal root ganglion through specific electrodes. The introduced energy will continue to act on the peripheral nerve or dorsal root ganglion in a manner of stimulating twice per second, which inhibits evoked synaptic activity, increases the threshold of pain tolerance, and reduces pain sensitization. PRF treatment only blocks nerve conduction and sensation; it is, thus, considered to be effective, safe, rapid, with few side effects, and the effect lasts for a long duration without damaging the nerve system [1,2,3,4,5,6,7]. Previous reports demonstrated that consistent nociceptive stimuli of different modalities, including mechanical, thermal, electrical, or chemical stimulation, can be applied to different tissues for differentiated and produced pain with various mechanisms [8,9]. Although PRF treatment is considered a safe strategy for pain management, there are still some complications that have been reported and have attracted attention [10,11]. Some complications are temporary and easily controlled, including local swelling and pain at the site of needle insertion, light-headedness, flushing, sweating, nausea, hypotension, and syncope. Serious complications may include neural trauma, hematoma formation, nerve injury [12,13], spondylodiscitis, intra-articular abscess, systemic infection, and meningitis. Furthermore, Cahana et al. pointed out that if the distance between the electrode tip and nerve cells is less than 500 μm during PRF treatment, it may cause damage to the cells, and these nerve tissue injuries often occur in the acute stage, although the acute effects of PRF may be reversible [14].

MAPKs, including p38 MAPK, ERK1/2, and JNK/SAPK, play critical roles in regulating neural plasticity and neuronal inflammation [15]. Evidence shows that ERK1/2, p38 MAPK, and JNK/SAPK promote pain sensitization after nerve damage through different molecular and cellular mechanisms. Phosphorylation of MAPK-related signaling pathways may activate non-transcriptional and transcriptional regulation, which results in the induction and maintenance of pain hypersensitivity under different pain conditions. Recent studies have revealed that nociceptive activity stimulates neurons in the spinal dorsal horn through multiple neurotransmitter receptors and regulates inflammatory pain sensitization-related gene transcription by activating p38 MAPK and ERK1/2 and regulating the activity of glutamate receptors and ion channels. When the nerve is injured, p38 MAPK and ERK1/2 are activated in microglial cells [16,17,18,19] and JNK/SAPK is activated in astrocytes [20,21] in the spinal dorsal horn, which may induce the production of proinflammatory cytokines and pronociceptive mediators, thereby subsequently enhancing and prolonging pain [22]. Accumulating evidence indicates that glial cells (microglial cells and astrocytes) in the spinal cord play critical roles in the pathogenesis of pain [23,24], and activation of MAPKs in glial cells is essential for the development and maintenance of neuropathic pain. Therefore, the activation of MAPKs plays an important role in increasing pain sensitivity regardless of the central or peripheral nervous system. It has also been demonstrated in different animal models that the inhibition of MAPK signaling pathways can alleviate inflammatory and neuropathic pain. The development of specific antagonists for MAPKs or inhibitors of MAPK-related pathways to target neurons and glial cells may lead to new strategies for pain management in the future.

Although PRF treatment can effectively improve refractory pain, once process accidentally on the intact nerve or unexpected situations occur during PRF treatment, the intact nerve may be injured, thereby triggering noxious pain. Although scholars in the previous demonstrated that the damage caused by PRF in the treatment process was temporary, while PRF treatment is widely used, few studies have elaborated on the fact that PRF stimulation may affect sensory nerves and dorsal root ganglion in terms of morphological modifications and nerve inflammation [12,13]. Therefore, in this study, we explored the analgesic effect of PRF under clinical application (waves with a 480 kHz frequency, output with 30–60 V, and pulse width of 20 ms; delivered for 6 min) on intact nerve function and in the activation of MAPKs in neurons and glial cells of the spinal cord.

## 2. Results

### 2.1. The Clinically Used Voltages of PRF Induced Mechanical Allodynia and Cold Hyperalgesia in Rat Paw

**Figure 1:**
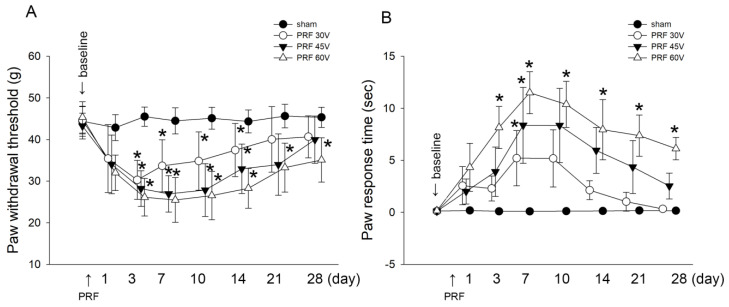
The paw withdrawal threshold and response time in the mechanical allodynia and cold hyperalgesia test. The behavioral and time response in rats (*n* = 8) subjected to sham (●) or 30 V (○), 45 V (▼), or 60 V (△) of PRF stimulation. (**A**) Mechanical allodynia was evaluated using the dynamic plantar esthesiometer. (**B**) Cold allodynia was evaluated by the acetone spray test. Sham: operated muscle. * *p* < 0.05 were compared with the sham group.

First, to explore the effect of the PRF voltage used clinically (30–60 V) on the function of the intact and naïve nerves, we stimulated the nerve with a PRF of 30–60 V, and then performed behavioral testing of tactile allodynia and cold allodynia. As shown in Figure 1A, after PRF application, the 30 V, 45 V, and 60 V of PRF stimulation induced a significantly greater analgesic effect compared with the sham group from Days 3 to 14. The algesic effect of the 30 V and 45 V of PRF was relieved on Day 21 after stimulation. However, after stimulation with PRF at 60 V, the algesic effect persisted until Day 28 after stimulation with no relief. Additionally, regarding cold hyperalgesia, 30 V and 45 V of PRF may induce a significantly greater nociceptive response and reach a maximum response compared with the sham group at Day 7 after stimulation; subsequently, the cold hyperalgesia may gradually be relieved. In contrast to 30 V and 45 V stimulation of PRF, 60 V of PRF stimulation may quickly trigger the nociceptive response on the third day after stimulation and reach the maximum value on the seventh day after stimulation; however, although the nociceptive response maybe gradually relieved on the seventh day after stimulation, it may delay to the twenty-eighth day and still not alleviate to the baseline. These results indicate that the PRF treatment commonly used in clinical practice still has the possibility of inducing pain, especially the most commonly used voltage of 60 V, which has a negative impact on nerve conduction function and can last the longest.

### 2.2. PRF Induced Inflammatory MAPKs Activation in Non-Spared Injury/Intact Nerve in the Lumbar Dorsal Horn

**Figure 2:**
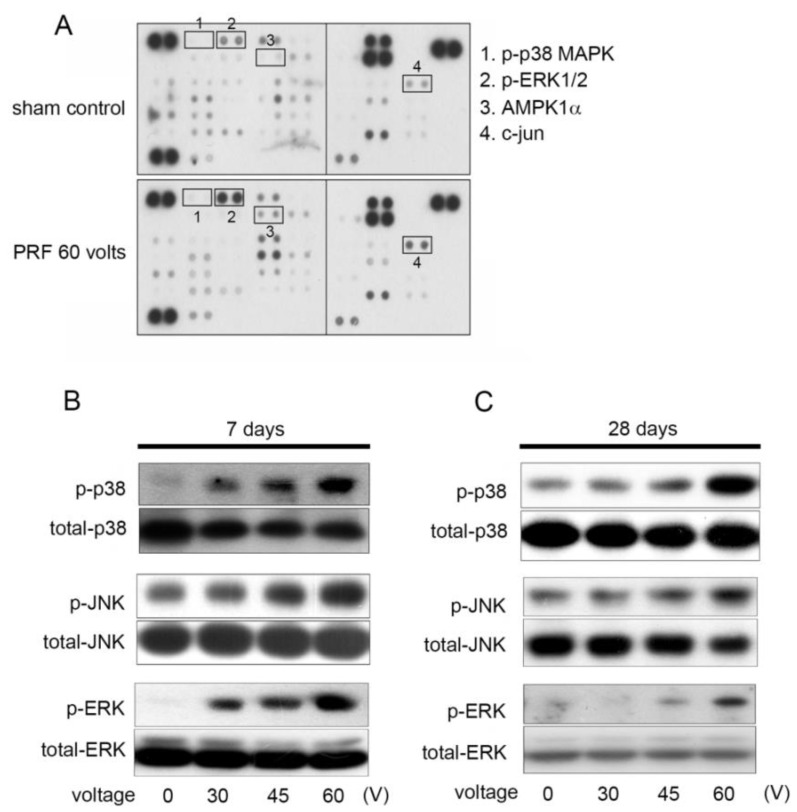
Phosphokinase activity assay and Western blot analysis of MAPKs activation in the lumbar dorsal horn of PRF-stimulated rats. (**A**) Template showing the location of specific antibodies (1, phosphor-p38 MAPK; 2, phosphor-ERK1/2; 3, AMPK1α; 4, c-jun) spotted onto the membrane. Each antibody was spotted in duplicate. The marked activations of phospho-MAPKs in response to 60 V of PRF stimulation are indicated by Arabic numerals. (**B**,**C**) Rats were stimulated with PRF at the indicated intensities (30 V, 45 V, and 60 V), followed by 7 or 28 days of rest. Then, the left dorsal horn of the rats was harvested and subjected to Western blot analysis. The phospho-p38 MAPK, phospho-ERK1/2, and phospho-JNK/SAPK were identified. The total-p38 MAPK, total-ERK1/2, and total-JNK/SAPK were used as a loading control.

Previous studies have shown that inflammatory responses can block nerve transduction and trigger neuropathic pain [15]. We speculate that the mechanical allodynia and cold hyperalgesia induced by the 60 V of PRF in rats may be caused by PRF triggering an inflammatory response in the lumbar dorsal horn. Therefore, phosphokinase activity assays and Western blotting were performed to identify MAPK activity. Based on the findings of the 60 V of PRF once stimulation induced persistent allodynic effects, we performed phosphoproteomic analysis using PRF-treated rats on Day 28. As shown in Figure 2A, the phosphokinase array chip demonstrated marked activation of phospho-p38 MAPK, phospho-ERK1/2, AMP-activated protein kinase α1 (AMPKα1), and c-Jun/transcription factor in response to the 60 V PRF stimulation. Furthermore, we used Western blotting to confirm the results presented by the phospho-kinase array chip, and analyzed the effect of different voltages of PRF stimulation on the activation of MAPKs in neurotransmission-related cells in the lumbar dorsal horn. In Figure 2B, the activation of phospho-p38 MAPK, phospho-ERK1/2, and phospho-JNK/SAPK was observed on Day 7 after PRF stimulation with both 30–60 volts. Figure 2C shows the activation of MAPKs on the 28th day after PRF stimulation. It clearly shows that phospho-p38 MAPK, phospho-ERK1/2, and phospho-JNK/SAPK were still activated. However, compared with Figure 2B, the activation of phospho-p38 MAPK, phospho-ERK1/2, and phospho-JNK/SAPK on Day 28 after 30 V and 45 V of PRF stimulation was almost similar to that without stimulation; the activation of MAPKs until the twenty eighth day after stimulation may be maintained only at 60 V of PRF. These results indicate that the PRF treatment commonly used in clinical practice still has the possibility of inducing the activation of inflammatory MAPKs such as p38 MAPK, ERK1/2, and JNK/SAPK. Additionally, the most commonly used 60 V of PRF can indeed induce a longer-lasting activation of MAPKs, and we speculate that maintaining the long-term activation of inflammatory MAPKs is related to long-lasting mechanical allodynia and cold hyperalgesia.

### 2.3. PRF Induced Inflammatory Cytokines and Matrix Metalloproteinases Expression in Non-Spared Injury/Intact Nerves of the Lumbar Dorsal Horn

**Figure 3:**
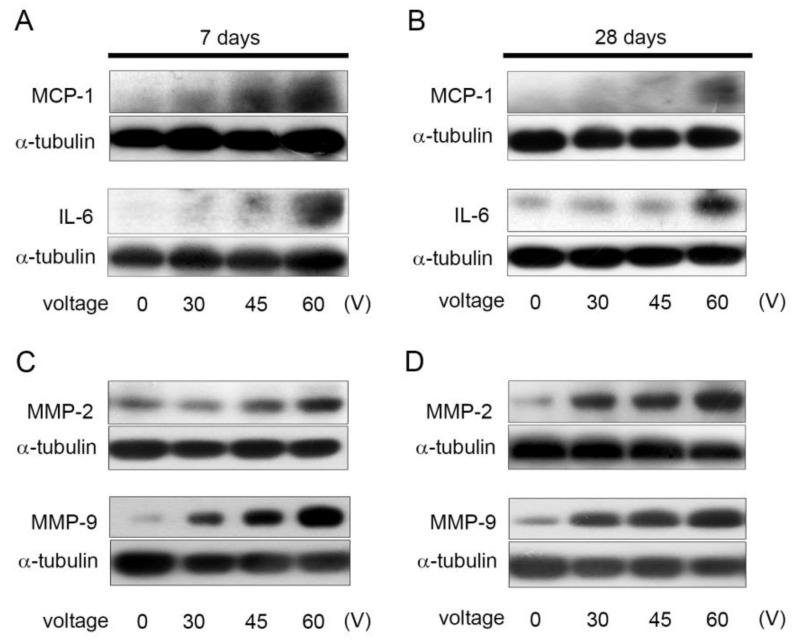
Effects of PRF treatment on the expression of pain-related cytokines and matrix metalloproteinases. Rats were stimulated with PRF at the indicated intensities (30 V, 45 V, and 60 V), followed by 7 (**A**,**C**) or 28 (**B**,**D**) days of rest. Then, the left dorsal horn of rats was harvested and subjected to Western blot analysis. MCP-1, IL-6, MMP-2, and MMP-9 were identified. α-tubulin was used as a loading control.

Cytokines are pluripotent proteins that play key roles in the induction and maintenance of pain [25]. In particular, monocyte chemoattractant protein (MCP)-1 increases the excitability of nociceptive neurons in chronically pathological dorsal root ganglia [25] and interleukin (IL)-6 plays critical roles in the development of pathological pain [26]; therefore, we wanted to analyze the MCP-1 and IL-6 expressions in the spinal cord after PRF stimulation. As shown in Figure 3A,B, 7 days after stimulation of the nerve with PRF 30–60 V, *MCP-1* expression increased in a voltage-dependent manner, and after 28 days of PRF stimulation with 30 V and 45 V, the expression of *MCP-1* returned to the baseline; however, rats stimulated with 60 V of PRF still had a high production of MCP-1 in the lumbar dorsal horn on Day 28. In contrast, for IL-6, PRF stimulation at 45 V only slightly increased on Day 7; if the voltage was increased to 60 V, it may increase the production of IL-6 on the seventh day after stimulation and maintain a high performance on the 28th day. Scientists currently believe that matrix metalloproteinases (MMPs) are important for the development and maintenance of the nervous system. MMP-2 and MMP-9 have been reported to contribute to the occurrence and persistence of neuropathic pain [27]. Therefore, we need to analyze whether mechanical allodynia and cold hyperalgesia in rats induced by PRF in this study are caused by the production of MMP-2 and MMP-9. The production of MMP-2 and MMP-9 in the lumbar dorsal horn was analyzed by Western blot analysis, and the results are shown in Figure 3C,D. There was no significant increase in MMP-2 on the seventh day after stimulation with 30 V PRF; when the PRF voltage was increased to 45 V for stimulation, a slight increase in MMP-2 was observed on the seventh day after stimulation, while 30 V and 45 V of PRF stimulation did not show a significant increase in MMP-2 until the 28th day; however, 60 V of PRF stimulation can make MMP-2 have an earlier increase in performance, showing a significant accumulation in the lumbar dorsal horn on Day 7. The production of MMP-9 was different from that of MMP-2. PRF stimulation at 30 V, 45 V, and 60 V rapidly increased the expression of *MMP-9* on the seventh day; the expression of *MMP-9* was also positively correlated with voltage, and it continued to accumulate until the 28th day. Based on the above results, we can speculate that the PRF treatment voltage commonly used in clinical practice may cause inflammation of nerve tissue and increase the expression of proteins and mediators related to neuropathic and inflammatory pain, including an increase in cytokines and MMPs. This also verified the fact that PRF stimulation increases mechanical allodynia and cold hyperalgesia in rats.

### 2.4. The 60 V PRF Treatment Commonly Used in Clinical Practice Induced the Activation of p38 MAPK and ERK1/2 in Neurons and Microglial Cells

**Figure 4:**
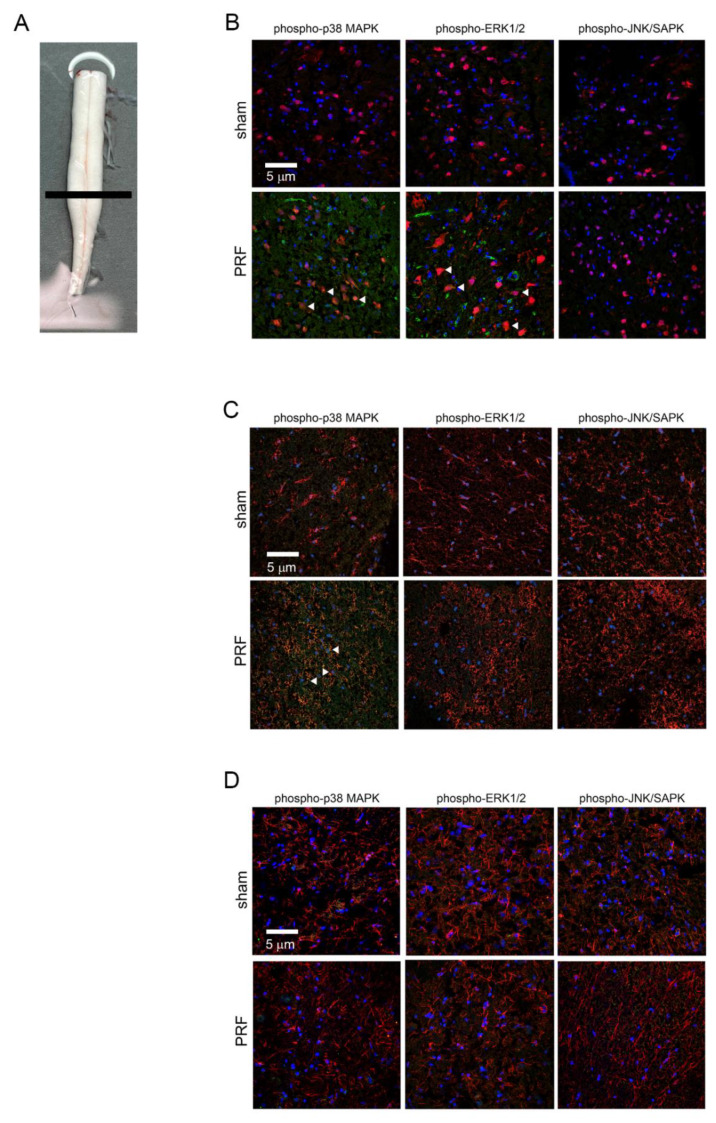
The spinal cords were obtained from Wistar rats that had undergone PRF stimulation for 28 days. The tissue photograph of spinal cord demonstrated the level of section (**A**). The spinal cord slides were stained using anti-NeuN, anti-CD11b, or anti-GFAP conjugated Rhodamine antibodies to identify neuron cells (**B**), microglial cells (**C**), and astrocytes (**D**), respectively (red). Additionally, the phosphorylated p38 MAPK, phosphorylated ERK1/2, and phosphorylated JNK/SAPK were stained by anti-phosph p38 MAPK, anti-phosph ERK1/2, or anti-phosph JNK/SAPK conjugated Alex 488 antibodies. The photo shows the dorsal horn of the spinal cord. The arrows indicate the phosph-p38 MAPK, phosph-ERK1/2, and phosph-JNK/SAPK expressions (green) in the Figure (**A**–**C**). The DAPI staining for the nucleus of the cells and photo presented in the magnification of 200×.

According to the results of Western blotting, 28 days after stimulation with 60 V PRF, the phospho-p38 MAPK, phospho-ERK1/2, and phospho-JNK/SAPK in rat lumbar dorsal horn still showed high performance and activation. Furthermore, p38 MAPK, ERK1/2, and JNK/SAPK are differentially activated in spinal glial cells (microglia and astrocytes are included) after nerve injury, which may lead to the synthesis of proinflammatory arbitrators, consequently enhancing and prolonging pain [15]. We want to clarify that the phospho-ERK1/2, phospho-p38 MAPK, and phospho-JNK/SAPK were expressed in the lumbar dorsal horn 28 days after stimulation with 60 V PRF, produced from neurons, microglial cells, or astrocytes. Therefore, in order to determine the activation of MAPKs in the dorsal horn cells of the spinal cord, including neurons, microglial cells, or astrocytes after stimulation by PRF, we used anti-NeuN, anti-CD11b, and anti-glial fibrillary acidic protein (GFAP) antibodies to target specific cells, respectively, and double immunofluorescent staining for phospho-p38 MAPK, phospho-ERK1/2, and phospho-JNK/SAPK. Figure 4B shows the activation of phospho-p38 MAPK and phospho-ERK1/2 in NeuN-positive neuronal cells. Compared with the sham group, the neurons of the lumbar dorsal horn in rats that received 60 V PRF stimulation had higher activities of phospho-p38 MAPK and phospho-ERK1/2 activity, but not phospho-JNK/SAPK. Figure 4C shows CD-11b positive microglial cells. PRF stimulation increased the expression of p-p38 MAPK in microglial cells; however, the expression of phospho-ERK1/2 and p-JNK/SAPK in the cells did not increase due to PRF stimulation. Finally, Figure 4D shows that the expression of phospho-p38 MAPK, phospho-ERK1/2, and phospho-JNK/SAPK did not significantly change in GFAP-positive astrocytes after PRF stimulation in the rats. The above-mentioned results suggest that PRF stimulation may induce inflammation-related MAPK activation in the lumbar dorsal horn, which mainly occurs in neurons and microglial cells, but not in astrocytes. We believe that the phosphorylation of p38 MAPK in neurons and microglial cells and ERK1/2 in neurons may play important roles in mechanical allodynia and cold hyperalgesia that still exist 28 days after rats are stimulated by 60 V of PRF. In contrast, PRF did not stimulate MAPK activation in astrocytes.

### 2.5. MAPK Antagonists Treatment May Relief PRF-Induced Mechanical Allodynia and Cold Hyperalgesia in Rats

**Figure 5:**
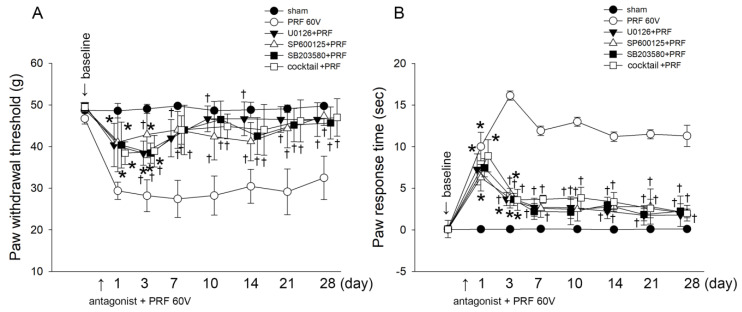
Comparison of the effects of MAPK antagonists on the paw withdrawal threshold and response time in the mechanical and cold allodynia tests. (**A**) Behavioral response and (**B**) paw response time in rats subjected to p38 MAPK antagonist (SB203580) plus PRF-60 volts (■), ERK1/2 antagonist (U0126) plus PRF-60 volts (▼), JNK/SAPK antagonist (SP600125) plus PRF-60 volts (△), antagonist cocktail (SB203580 + U0126 + SP600125) plus PRF-60 volts (□), sham operation (●), or PRF-60 volts (○). * *p* < 0.05; PRF-60 volts, U0126 plus PRF-60 volts, SP600125 plus PRF-60 volts, SB203580 plus PRF-60 volts, and antagonist cocktail plus PRF-60 volts compared with the sham group. † *p* < 0.05; sham, SB203580 plus PRF-60 volts, U0126 plus PRF-60 volts, SP600125 plus PRF-60 volts, and antagonist cocktail plus PRF-60 volts groups compared with the PRF-60 volts group.

Figure 1 shows that 60 V of PRF induced mechanical allodynia and cold hyperalgesia in rat paws; Figure 3 and Figure 4 show that PRF increased spinal cord activation of p38 MAPK, ERK1/2, and JNK/SAPK. Therefore, we used p38 MAPK, ERK1/2, and JNK/SAPK antagonists, respectively, or cocktailed to treat PRF-stimulated rats and observe improvements in mechanical allodynia and cold hyperalgesia. The results of mechanical allodynia in rats from the dynamic plantar esthesiometer are shown in Figure 5A. Both p38 MAPK antagonist (SB203580), ERK1/2 antagonist (U0126), JNK/SAPK antagonist (SP600125), and the cocktail of all antagonists (SB203580 + SP600125 + U0126) treatments resulted in a significant relief of pain in the rats from Day 3 to 28 during the entire observation period, in spite of the nociceptive behaviors in the rats from Day 1 and 3 after the 60 V PRF stimulation. Similar mechanical allodynia and cold hyperalgesia after the acetone spray test are presented in Figure 5B. SB203580, U0126, SP600125, and a mixture of all antagonists had a significant potential to relief cold hyperalgesia in rats from Day 3 to 28 during the entire observation period. These results clearly show that MAPK antagonists can effectively alleviate the pain caused by 60 V PRF stimulation. This also means that PRF can increase mechanical allodynia and cold hyperalgesia in rats by activating MAPKs in nerve cells. These results clearly show that MAPK antagonists can effectively alleviate the pain caused by 60 V of PRF stimulation. It also means that PRF can increase mechanical allodynia and cold hyperalgesia in rats by activating MAPKs in the nervous system.

## 3. Discussion

### 3.1. Application, Comorbidity, and Impact of PRF for Pain Management

PRF treatment for pain management was successfully developed in 1995, and it was derived from radiofrequency (RF) treatment [28,29,30]. RF treatment mainly destroys nerve tissue through the heat source generated by a high-frequency current to effectively control pain; however, RF often damages normal nerves and surrounding tissues, causing complications. Clinicians still have many doubts regarding its application; PRF is a high-frequency current applied in the form of pulses and short pulses to block pain signals, and it is less likely to damage nerves due to high temperatures. The principle of PRF application is to achieve pain relief without permanent nerve damage. PRF treatment can provide effective pain relief for the dorsal root ganglion (DRG) of the cervical and lumbar spine in a safe strategy environment [31,32], and PRF treatment also provides effective relief for trigeminal neuralgia [33]. Although the therapeutic benefit of PRF has been recognized by the public, and its safety is higher than that of RF, there is still the possibility of developing complications in the process of its clinical application [14]; therefore, this study analyzes the possible negative effects of PRF on intact nerves. In our study, we found that PRF stimulation of intact nerves causes nerve injury; particularly, 60 V of PRF may cause mechanical allodynia and cold hyperalgesia in rats. We found that the rats did not recover from the effect of 60 V of PRF on pain behavior compared with 30 V and 45 V as well as sham groups. The pain lasted 28 days regardless of the mechanical or thermal allodynia. We do not know whether the pain induced by 60 V of PRF and the inflammatory response to nerves are irreversible or how long it lasts, but we can clearly state that this stimulus is very strong and sufficient to damage nerve function. Therefore, based on these results, we provide recommendations as a reference for applying PRF treatment to refractory pain for pain management specialists. We suggest that when using PRF to manage intractable pain, if the expected effect cannot be achieved or the patient claims that the treatment has worsened the pain, the pain management specialist should consider the accuracy of the diagnosis, the accuracy of the target nerve to be treated, and the possible damage to intact nerves resulting from PRF stimulation.

Human sciatic nerves are derived from L4–L5, S1–S3. They merge to form a trunk-like nerve, sciatic nerve. After the trunk extends downward, it will branch into tibial nerve and common fibular nerve. There are four branch nerves in front of tibial nerve. They dominate the adductor magnus muscle, semitendinosus muscle, semimembranosus muscle, and the long head of the biceps femoris muscle. The tibial nerve and common fibular nerve have multiple branches distributed in the lower limbs. When the patient’s sciatica pain occurs, the clinician can use dermatomes to distinguish the single nerve where the pain occurs, and then PRF treatment can be performed at the appropriate proximal site of nerve root which close to dorsal root ganglion. In general, clinical PRF treatment is rarely performed directly on the sciatic nerve. Although a few treatments must be performed on the downstream of sciatic nerve, clinicians still need to accurately administer the target nerve to avoid unnecessary nerves injury and pain. Although the purpose of this study is to illustrate the possible impact and damage of PRF on the intact nerve, which is slightly different from the concept of clinical use of PRF to treat chronic pain. We still believe that the results obtained by using PRF to stimulate the sciatic nerve of rats can still remind the importance of precision in the target nerve when administering PRF treatment for physicians, although it is often clinically applied to the proximal site of dorsal root ganglion.

### 3.2. Correlation between Inflammatory and Neuropathic Pain

In general, chronic pain can be categorized into inflammatory pain and neuropathic pain; the former is caused by nociceptive stimulation and a series of inflammatory processes; the latter is caused by neuroplastic changes and causes hypersensitivity in peripheral and central nociceptive systems [34]. Inflammation can contribute to tissue pressure and dysfunction [35], and toxic substances and thermal stimuli around nociceptive receptors can also induce inflammatory pain [36]. The nociceptive signals that occur in the central nervous system can cause pain perception to the nerves, and the brain’s perception of pain can regulate and maintain the stability and coordination of the immune, autonomic, and endocrine systems. An imbalance in these signaling pathways can lead to inflammatory pain [35]. Recently, we demonstrated that injury induced pain and the subsequent neuroinflammation may have been the crucial factors influencing depression-like behavior in animals [37]. Inflammatory cytokines are triggered to induce inflammatory responses in the surrounding sites of nerve injury [36]. In fact, when peripheral nerve inflammation continues to occur, it can be observed that the marker-growth associated protein 43 (GAP43) and activating transcription factor 3 (AT3) performance of neuropathic pain in the DRG will increase [38,39], indicating that the inflammatory response can cause neuronal damage and neuropathic pain. Subsequently, neuronal injury can cause neuronal inflammation and induce secondary inflammatory pain. Both pains influence each other and often occur together. Damage to the surrounding tissues and nerves increases pain. The causes of pain include DRG neuron sodium channels [40], potassium channels [41,42], or voltage-gated calcium channels [43,44] change in expression; increased release of glutamate from primary afferent neurons in the dorsal horn; enhanced glutamate receptor function of second neurons; and increased astrocyte and microglial cell activation in the dorsal horn [45,46]. It is interesting that as the time of tissue damage increases, many changes in the dorsal horn ganglion and dorsal horn are similar to those observed after nerve damage. Although the timing of each mechanism and factor may be different, inflammatory pain and neuropathic pain share the same mechanism. Therefore, during neuropathic pain management, a possible inflammatory response and inflammatory pain should be considered.

### 3.3. The Role of MMPs, MCP-1, IL-6 in Pathological Pain and Nerve Inflammation

MMPs play a very important role in inflammatory and neuropathic pain, especially MMP-2 and MMP-9. Patients with Alzheimer’s disease, multiple sclerosis, amyotrophic lateral sclerosis, stroke, spinal cord injury, or epilepsy are known to have increased MMP-9 and MMP-2 levels in the central nervous system [47,48,49,50,51]. MMP-9 and MMP-2 can decompose the extracellular matrix in the central nervous system, destroy cell–cell and cell–matrix homeostasis, damage the barrier function of the blood–brain barrier, induce vascular leakage and tissue edema, and trigger neuron apoptosis. In the process of chronic neurological dysfunction remodeling, MMP-2 and MMP-9 [52,53] induce the occurrence of abnormal neural circuits, which is very important for the development of neuropathic pain [50]. A previous study has shown that sciatic nerve injury may increase the expression of MMP-9, which in turn degrades myelin basic protein and leads to nerve demyelination [54]. In the investigation of spinal cord ligation in mice, it has been shown that MMP-9 rises rapidly and continues to be produced in large amounts. Intrathecal injection of tissue inhibitors of metalloproteinase-1, MMP-9 siRNA, or MMP-9 antagonist can effectively inhibit the occurrence of mechanical allodynia in the early stage of injury [55]. In addition, after nerve injury, sensory neurons produce spontaneous discharge and release MMP-9, and this MMP-9 will activate the IL-1β precursor protein-pro-IL-1β, which is increased due to the damage and stimulation of microglial cells. MMP-9 in soma cells of the DRG is also transported to the dorsal horn to activate microglial cells and work with IL-1β to induce early neuropathic pain [55]. MMP-9 induces the activation of pro-IL-1βduring the early stages of neurological injury. Additionally, MMP-2 is also related to hyperalgesia after nociceptive stimulation and is also important for the activation of spinal microglial cells; however, MMP-2 activates IL-1β and stimulates microglial cells in the late stage of nociceptive stimulation to promote the development of advanced neuropathic pain [55], and its effect and occurrence are mostly related to the occurrence of late-stage neuropathic pain [55].

Chemokines are closely related to the occurrence of inflammatory, degenerative, and nociceptive neurological diseases, whether in the central or peripheral nervous system, especially MCP-1,which plays the most important mediator role [56]. MCP-1 regulates the activation and proliferation of microglial cells, thereby promoting inflammatory responses in the central nervous system [57]. MCP-1 also increases the integrins of monocytes, which in turn tightly connects leukocytes and endothelial cells [58], and regulates endothelial adhesion molecules and cytokine production to increase the permeability of the blood–brain barrier [59]. MCP-1 also participates in the regulation of hyperalgesia through direct interaction with sensory neurons and indirectly through leukocyte activation in the peripheral nervous system [60,61]. Scientists have demonstrated a large increase in MCP-1 in damaged sensory neurons, DRG, and Schwann cells [62]; in addition to mediating the inflammation of DRG, MCP-1 induced by cells in the DRG may also be involved in the signal transduction of pain response, including loss of nerve fibers and mechanical hypersensitivity. It has also been confirmed in animal experiments that *MCP-1* gene-deficient mice do not experience neuropathic pain caused by nerve injury [60,61]; MCP-1 is derived from cells in the neuronal system after nerve injury, and is mainly involved in the pathogenesis of neuropathic pain [56]. Understanding and effectively regulating neuroinflammation mediated by MCP-1 may be used as a new management strategy for neuropathic and inflammatory pain.

Accumulating evidence indicates that IL-6 is an emerging regulator of pathological pain, which plays an important role in the pathogenesis of neuropathic pain. In a rat model experiment, it was found that the dorsal and ventral horn of the spinal cord had increased IL-6 mRNA and IL-6 protein levels after nerve injury [63,64]; intrathecal injection of IL-6 can also increase mechanical allodynia after sciatic cryoneurolysis in rats, and it can be relieved by injection of IL-6 antibody [65]. In addition, it can inhibit the expression of IL-6 by increasing the activation of cannabinoid CB2 receptors, thereby inhibiting hyperalgesia due to the sensitization of primary nociceptive neurons [66]. In addition, clinical trials have also found that patients with lumbar spinal stenosis-induced sciatica injected with anti-IL-6R monoclonal antibody (tocilizumab) via epidural injection can effectively reduce radicular leg pain and numbness [67]. In fact, studies have also shown that nerve injury increases the production of IL-6 in nerves and surrounding tissue. In vitro and animal experiments have shown that prostaglandin E2 (PGE2) and IL-6 released by damaged nerve cells and neurons in the DRG are significantly elevated, and elevated PGE2 and IL-6 can be inhibited by COX2 inhibitors, L-161982 (a selective PGE2 receptor 4 antagonist) and calphostin C (a protein kinase C inhibitor) [68]. Additionally, we recently demonstrated that uptake ratios of serotonin transporters were significantly lowered in the thalamus and striatum of rats brain with nerve injury-induced neuropathic pain and depression-like behaviors. Additionally, the decrease in serotonin transporters density was associated with the pronounced IL-6 production [69]. In short, nerve injury can lead to an increase in the expression of IL-6, and IL-6 is also an important mediator of both neuropathic and inflammatory pain.

### 3.4. The Role of Mitogen-Activated Protein Kinase Signaling in Pain

Current research shows that MAPKs, including p38 MAPK, ERK1/2, and JNK/SAPK, play a key role in the occurrence of chronic nociceptive sensitization [15,70]. Neurotransmitters, growth factors, hormones, and cytokines may activate the nervous system and increase nerve excitability [71]. The interaction between neurons, microglial cells, and astrocytes plays the role of a commander in the development of pathological pain. The intracellular phosphorylation of MAPKs in DRG, neurons in the spinal cord and cortex, microglial cells, and astrocytes are the switches of the signaling pathways of pain conduction; therefore, controlling MAPKs is considered to have the potential to become a pain control strategy. The activation of p38 MAPK in microglial cells of the spinal cord is related to the generation of inflammatory pain [72] and motor fiber injury-induced neuropathic pain [73], and it has been shown in many different research models that after spinal nerve [16,17,26] and sciatic nerve [74] ligation, spared nerve injury [75], spinal cord injury [25], and intracellular p38 MAPK in microglial cells of the spinal cord dorsal horn is rapidly activated. After nerve injury, the increase in TNF-α, IL-1β, MCP-1, inducible NOS, cathepsin S, COX-2, and MMP-9 are all factors that stimulate the activation of p38 MAPK in microglial cells [15]. p38 MAPK also promotes the activation of phospholipase A2 by activating its downstream MAPKAP kinase-2. The activation of Phospholipase A2 leads to the production of arachidonic acid and prostaglandin, which are mediators that cause neuroinflammation and inflammatory pain; p38 MAPK plays a central role in coordinating excitatory neuron and neuroglia damage, regulating synaptic function, and the signal transduction of pain response. Numerous studies also point to the fact that inhibiting the activity of p38 MAPK in nerve tissue can effectively eliminate neuropathic and inflammatory pain caused by various injuries [15]. Additionally, intraplantar injection of capsaicin and ligation of the spinal nerve may induce the phosphorylation of ERK1/2 in nerve fibers [76], neurons, microglial cells, and astrocytes [18], and subsequently induce mechanical allodynia and neuropathic pain. Neurons in the dorsal horn of the spinal cord by nociceptive stimulation may activate ERK1/2, induce gene transcription through neurotransmitter receptors and second messenger pathways, and play a key role in central sensitization. In the central nervous system, inflammatory pain sensitization also requires activation of ERK1/2 in amygdala neurons, which activates ERK1/2 in microglial cells and astrocytes, leading to the synthesis of proinflammatory/pronociceptive mediators, thereby enhancing and prolonging pain [15]. In the process of nerve injury, the activation of JNK/SAPK is different from that of p38 MAPK and ERK1/2; the activation of JNK/SAPK after sciatic nerve injury is mostly seen in spinal astrocytes [21], and the activation of JNK/SAPK signaling is important for the development and maintenance of neuropathic pain. Intrathelial injection of a JNK/SAPK inhibitor (SP600125) and an inhibitory peptide (D-JNKI-1) may alleviate neuropathic pain [21,77]. When nerves are stimulated, both fibroblast growth factor-2 (FGF-2) and TNF-α will increase; TNF-α activates JNK/SAPK in astrocytes rapidly, and FGF-2 plays an important role in the continuous activation of JNK/SAPK and the maintenance of pathological pain [15]. Astrocytes in the spinal cord show that MCP-1 increases after peripheral nerve injury; it is transmitted and induces central nervous system sensitization by enhancing the excitatory synapses of neurons in the dorsal horn. The expression of MCP-1 in astrocytes is also regulated by the TNF-α-JNK/SAPK signaling axis. In addition, in spinal nerve ligation rats, IL-6 activates the JAK/SAPK-STAT3 signaling pathway in microglial cells of the spinal dorsal horn [78]; inhibition of STAT3 activity can reduce mechanical allodynia and thermal hyperalgesia in rats. Activation of JAK/SAPK is involved in the development of neuropathic pain. In conclusion, inhibition of all p38 MAPK, ERK1/2, and JNK/SAPK signaling pathways has been shown to control inflammatory and neuropathic pain. The advancement of certain preventions for the MAPK pathways of target neurons and glial cells might lead to new strategies for pain management.

### 3.5. The Cellular and Molecular Mechanisms of PRF Therapy on Refractory Pain

PRF can be applied to a variety of refractory pain management, such as axial pain, lumbosacral or cervical radicular pain, trigeminal neuralgia, miscellaneous pain syndromes, etc. However, there are still not enough reports to describe the complete cellular and molecular mechanisms of PRF for the management of refractory pain. Previously, van Zundert et al. demonstrated that PRF can change the pathway of pain by regulating/increasing the expression of an immediate early gene, *c-Fos*, thereby inhibiting the occurrence of pain [79]. In contrast, the research of Higuchi et al. showed different results [80]. Therefore Richebe et al. did not believe that the efficacy provided by PRF is related to the expression of *c-Fos* [81]. Additionally, an indicator of cellular stress, activating transcription factor 3 (ATF3) is another factor of concerned. Scientists believe that PRF applied to dorsal root ganglia can cause small-diameter C and Aδ fibers to selectively increase of ATF3 [82]. PRF can provide an alteration in synaptic transmission and a neuromodulatory-type effect to achieve the purpose of inhibiting the transmission of pain signals [83]. PRF actually enhances the activation of regulatory pathways related to neuropathic pain, such as the descending noradrenergic and serotonergic inhibitory pathways [84]. PRF has biological effects on cell morphology, synaptic transmission and pain signals. In fact, electric treatment has been proven to affect the immune regulation of cells. It can reduce the production of TNF-α and IL-6 in degenerative lumbar spinal disorders [85,86], and increase the density of adenosine A2A receptor of neutrophils [87], that can inhibit the production of TNF-α, IL-6 and IL-8 [88]. We also demonstrated that the ERK1/2 activation and IGF2 production in the ipsilateral spinal dorsal horn of spared nerve injury rats were effectively inhibited after PRF treatment [89,90]. These studies are all fragmented about the mechanism of action of PRF, and more studies are needed to connect them in order to know the full picture of the related mechanisms of PRF for refractory pain management.

In this experiment, the rat was stimulated by 60 V PRF, and the results of Western blotting analysis showed that the activation of p38 MAPK and ERK1/2 in the dorsal horn of the spinal cord increased, and immunofluorescence was performed with tissue sections of the dorsal horn of the spinal cord (28 days) and compared with the results of Western blotting on Day 28. We speculate that PRF mainly activates p38 MAPK activation in neurons and microglial cells, and the activation of ERK1/2 is only seen in neurons. Activation of JNK/SAPK was not observed in neurons and glial cells after PRF stimulation for 28 days. These results are consistent with previous research results showing that physical damage to peripheral nerves or stress stimulation increases the activation of p38 MAPK in neurons and microglial cells of the central nervous system, as well as ERK1/2 activation in neurons. In addition, the JNK/SAPK antagonist effectively reversed mechanical allodynia and cold hyperalgesia (Figure 5) caused by PRF stimulation. However, when JNK/SAPK does not activate neurons or glial cells (Figure 4), we speculate that it may lead to the activation of JNK/SAPK occurring in the early stage (7 days) of PRF stimulation (Figure 2). Although ERK1/2 activation in microglial cells and p38 MAPK and ERK1/2 activation in astrocytes did not occur, which was different from results of some previous studies, we speculate that this is due to the different patterns of stimuli, and the damage is caused by different intracellular signaling pathways in the nervous system. Although the activation of MAPKs caused by 60 V of PRF on the naïve nerve was partially different from previous studies, the mechanical allodynia and cold hyperalgesia in the animals were obvious. Therefore, we believe that the clinical application of PRF induces nerve inflammation. This result suggests that pain management specialists should use the PRF strategy cautiously and pay attention to its possible harm.

### 3.6. Limitation of This Study

In clinic, the clinician can use dermatomes to distinguish the single nerve where the pain occurs, and then PRF treatment can be performed at the appropriate proximal site of dorsal root ganglion. In general, clinical PRF treatment is rarely performed directly on the sciatic nerve. However, animal studies in the laboratory often apply PRF directly to the sciatic nerve, instead of directly applying it to the dorsal root ganglion of spinal nerve. The main reason is that the rats have insufficient working space at the spine, and it is not easy to achieve the purpose of PRF stimulation. Therefore, whether this conclusion can truly reflect the phenomenon of clinical application of PRF deserves further study.

## 4. Materials and Methods

### 4.1. Animal Grouping and Treatment

Male Wistar rats (BioLASCO, Taipei, Taiwan) weighing 200–250 g were housed individually with soft bedding in a 12 h night/day cycle with free access to food and water at all times in a similar environment for 7 days, for acclimation before PRF. All efforts were made to minimize the number of animals used and their suffering. Rats were randomly divided into nine groups, and eight rats were included in each group. Group 1 (sham control, electrode but no output) consisted of rats that were fed a normal chow diet and did not received PRF; Group 2 (PRF-30 V) consisted of rats that received 30 V of PRF stimulation; Group 3 (PRF-45 V) consisted of rats that received 45 V of PRF stimulation; Group 4 (PRF-60 V) consisted of rats that received 60 V of PRF stimulation; Group 5 (p38 MAPK antagonist + PRF-60 V) consisted of rats that received SB203580 (50 mg/kg) treatment plus 60 V of PRF stimulation; Group 6 (ERK1/2 antagonist + PRF-60 V) consisted of rats that received U0126 (20 mg/kg) treatment plus 60 V of PRF stimulation; Group 7 (JNK/SAPK antagonist + PRF-60 V) consisted of rats that received SP600125 (20 mg/kg) treatment plus 60 V of PRF stimulation; Group 8 (cocktail of antagonist + PRF-60 V) consisted of rats that received SB203580, U0126, and SP600125 mixture treatment plus 60 V of PRF stimulation; Group 9 (DMSO + PRF-60 V) consisted of rats that received normal saline treatment plus 60 V of PRF stimulation. The optimal administration dosages of antagonists were selected according to previous studies [91,92,93,94]. The ERK1/2, JNK/SAPK, and p38 MAPK antagonists were all soluble in dimethyl sulfoxide (DMSO) and were obtained from Sigma-Aldrich Co., USA. These antagonists or vehicle were administered once by intraperitoneal injections 30 min before the 60 V PRF application.

### 4.2. Pulsed Radiofrequency Treatment

According to previous studies [89,90], PRF was administered via an electrocautery disk placed in the right decubitus position and connected to the PRF generator (NT1000; NeuroTherm, Hillbrow Liss, Hampshire, UK). The 5-mm and 22-gaugeactive tip electrodes were placed vertically adjacent to the left sciatic nerve (3–4 mm proximal to the treatment site). PRF treatment at 480 kHz of stimulation mode with an output of 30, 45, or 60 voltages was delivered at a rate of 2 Hz, 2 bursts/s with a 30 ms duration for 6 min (3 min per session, with a 10 s intersession interval) at a temperature below 42 °C.

### 4.3. Behavioral Testing of Tactile Allodynia and Cold Allodynia

Mechanical allodynia and cold allodynia were evaluated using a dynamic plantar esthesiometer (DPA; Ugo Basile, Comerio, Italy) according to a previously described procedure [89,90]. For screening of responsive allodynia, each rat was positioned in a private plastic cage with a wire mesh floor, seasoned to the cage for 15 min prior to each test session, and a paw withdrawal action was evoked by applying a boosting force using a blunt-end metal filament (diameter, 0.5 mm) focused on the territory of the sural nerve at the palmar surface of the left ipsilateral hind paw. The force was increased from 1 to 50 g in steps of 1 g over 20 s, and was then held at 50 g for an additional 10 s; the price of the force increase was 2.5 g/seconds. The threshold was tape-recorded as the force that elicited the hind paw removal reflex (the mean of three measurements carried out at 1-min periods).

Cold allodynia was determined by measuring the cold withdrawal feedback of the back paw to an acetone spray. Rats were placed in a clear plastic cage on top of a wire mesh grid, which permitted access to the paws, and were adjusted to the testing setting for 15 min prior to the dimension. Cold allodynia was assessed by spraying acetone (100 μL) using an Eppendorf multipipette onto the palmar surface area of the ipsilateral hind paw via the cord mesh flooring, and the duration of the shaking, flinching, attacking, or licking behavior that followed in a 1-minperiodwas measured. Each rat was examined five times at a marginal interval of 5 min. A minimal value of 0.5 s was given if there was a fast or vigorous reaction, whereas a value of 0 was given if no response was observed in all. Behavioral testing was performed 1 day before surgery (baseline) if there was no clinical evidence of nerve damage or various experimental days after PRF.

### 4.4. Mitogen-Activated Protein Kinase Activity Array

Rats were treated with or without PRF (60 V). On Day 28 after the single PRF stimulation, the lumbar dorsal horn (LDH) of the spinal cord was dissected. Tissues were lysed in ice-cold RIPA buffer containing protease and phosphatase inhibitors (Sigma-Aldrich, St. Louis, MO, USA). Protein concentration was determined using the Pierce BCA Protein Assay Kit (Thermo Fisher Scientific Inc., Waltham, MA, USA). Phospho-kinase array (R&D Systems, Minneapolis, MN, USA) was performed according to the manufacturer’s instructions. Briefly, the tissue lysates (600 μg) were mixed with array buffer and incubated with pre-blocked array membrane at 4 °C overnight. Membranes were then washed and probed with a primary antibody cocktail for 2 h, followed by a secondary antibody for 30 min. The membranes were then incubated with horseradish peroxidase-conjugated IgG. Immunodetection was performed using a chemiluminescence reagent following exposure to a ChemiDoc-It TM Imaging System (UVP, Upland, CA, USA).

### 4.5. Western Blotting

The rats were treated with PRF at the indicated voltages. At the indicated time points, the LDH of the spinal cord was dissected. Each sample was homogenized in ice-cold radio immunoprecipitation assay buffer in the presence of protease and phosphatase inhibitors. Equal amounts of total protein were resolved by SDS-PAGE and transferred to polyvinylidene fluoride membranes (Millipore, Bedford, MA, USA). After blocking for 1 h at room temperature in Tris-buffered saline containing 0.05% Tween 20 (TBST) and 5% skim milk, the membranes were incubated with anti-phospho-p38 MAPK (Santa Cruz, Dallas, TX, USA), anti-p38 MAPK (Santa Cruz, Dallas, TX, USA), anti-phospho-ERK1/2(Santa Cruz, Dallas, TX, USA), anti-ERK1/2 (Santa Cruz, Dallas, TX, USA), anti-phospho-JNK/SAPK (Santa Cruz, Dallas, TX, USA), anti-JNK/SAPK (Santa Cruz, Dallas, TX, USA), anti-MCP-1 (Thermo Fisher Scientific, Waltham, MA, USA), anti-IL-6 (R&D Systems, Minneapolis, MN, USA), anti-MMP-2 (Abcam, Waltham, MA, USA), and anti-MMP-9 (Abcam, Waltham, MA, USA) antibodies, followed by probing with a secondary antibody for 30  min. The membranes were then incubated with horseradish peroxidase-conjugated IgG. Immunodetection was performed using a chemiluminescence reagent following exposure to a ChemiDoc-It TM Imaging System (UVP, Upland, CA, USA).

### 4.6. Immunofluorescent Staining

Ten-micrometer sections from rat L4–L6 spinal cords were fixed in 4% paraformaldehyde for 10 min and permeabilized with 0.1% Triton X-100 in phosphate-buffered saline for 35 min, endogenous peroxidase activity was quenched using 3% H_2_O_2_ for 30 min, and non-specific binding sites were blocked with blocking solution (Vector Laboratories, Burlingame, CA, USA). Briefly, the slides were stained using anti-phospho-ERK1/2 conjugated Alex 488 (Santa Cruz Biotechnology, Dallas, TX, USA), anti-phospho-JNK/SAPK conjugated Alex 488 (ThermoFisher Scientific, Waltham, MA, USA), and anti-phosphor-p38 MAPK conjugated Alex 488 (Thermo Fisher Scientific, Waltham, MA, USA) antibodies. Slides of the spinal cord were stained using anti-NeuN (EMD Millipore, Billerica, MA, USA), anti-CD11b (GeneTex, Alton Pkwy Irvine, CA, USA), and anti-GFAP (EMD Millipore, Billerica, MA, USA) conjugated rhodamine antibodies to identify neuron cells, microglial cells, and astrocytes, respectively. The cellular nuclei were identified using4′,6-diamidino-2-phenylindole (DAPI).

### 4.7. Statistical Analysis

All data are expressed as the mean ± standard error of the mean (SEM). Statistical analysis was performed using one-way analysis of variance, followed by the least significant difference (LSD) post hoc test or Dunnett’s test for comparison of multiple groups. Statistical significance was set at *p* < 0.05.

## 5. Conclusions

These preliminary data suggest that the application of a clinical dose of 60 V of PRF stimulation may induce inflammatory pain, which correlates with the expression of MAPK activation and inflammatory mediators such as MCP-1, IL-6, MMP-2, and MMP-9 within the dorsal horn of the spinal cord in rats. The underlying mechanism most likely involves the MAPK pathway in neuronal and microglial cells. In addition, the results of this study also suggest that clinicians should consider the accuracy of the technical application when using the PRF strategy for pain treatment in order to avoid unnecessary damage and irritation to intact nerves.

## Data Availability

Not applicable.
